# Supplementary material for: Are carbon emissions trading and green financial instruments synergistic? -Comprehensive quantitative research based on content analysis
Source: PLoS One. 2024 Mar 7;19(3):e0298601. doi: 10.1371/journal.pone.0298601 (PMC10919601; doi:10.1371/journal.pone.0298601)
Supplement: S1 Data — (ZIP) [file pone.0298601.s001.zip › Data/地级市Z_F工作报告.zip!/║╙─╧/╚2├┼╧┐╩╨/└┤╫╘╣1⁄2╓┌║┼í╛┬φ┐╦╩2╛▌═°í┐_2007.docx]

各位代表：

现在，我代表市人民政府，向大会作政府工作报告，请予审议，并请各位政协委员提出意见。

一、本届政府工作回顾

本届政府任期是实施“十五”计划、开启“十一五”规划的重要时期。六年来，市政府在省委、省政府和市委的正确领导下，在市人大、市政协的监督支持下，牢牢把握“加快发展，维护稳定，狠抓落实”的工作大局，紧紧围绕跻身中原崛起第一梯队、提前实现全面建设小康社会的奋斗目标，全面落实科学发展观，积极构建和谐社会，团结奋进，扎实工作，圆满完成了市四届人大确定的各项目标任务。

——综合实力显著增强。国民经济连续六年保持较高的增长速度和质量，地区生产总值年均增长11.9%。2006年，地区生产总值完成411.85亿元，比上年增长16.5%；人均GDP突破1.8万元，居全省第五位。地方财政一般预算收入达21.63亿元，是2001年的3.5倍，比上年净增5亿元，增长30.1%。

——经济结构不断优化。三次产业比例为8.9∶63.7∶27.4，二、三产业比重91.1%，比2000年提高4.5个百分点。工业主导地位更加突出，去年全市工业增加值完成246.44亿元，是2000年的3.3倍。经济质量和效益明显提高，规模以上工业利税、经济效益综合指数连年位居全省前列，单位生产总值能耗有所下降。非公有制经济增加值达到165亿元，占全市经济总量的40%。

——基础设施更加完善。六年累计完成固定资产投资713.6亿元，是“九五”时期的3.06倍。新增公路通车里程966公里，发电装机容量新增200万千瓦。中心城市“一城三区”框架基本形成，相继获得中国优秀旅游城市、中国特色魅力城市、全国水土保持生态环境建设示范城市称号。全市城镇化率达到40.8%，高出全省8.3个百分点。

——改革开放深入推进。国有企业改革基本完成，重点领域改革进一步深化。2006年，直接利用外资1.38亿美元，比上年增长91.8%，绝对值居全省第三位，增幅第一位；出口创汇完成1.52亿美元，增长9.7%。六年累计实际直接利用外资3.5亿美元，是“九五”时期的3.6倍；累计实现出口创汇6.2亿美元，是“九五”时期的9.8倍。

——社会事业全面进步。科技进步对经济增长的贡献率为50.4%，比2000年提高9.3个百分点。各级、各类教育事业全面发展，“普九”提高目标顺利实现，文化、体育事业日益繁荣。城乡卫生条件不断改善，六年累计改造乡镇卫生院60所，疾病预防控制体系逐步健全。人口自然增长率年均控制在4.37‰以内，人口素质显著提高。

——人民生活较大改善。2006年，城镇居民人均可支配收入9072元，六年平均增长9.6%；农民人均纯收入3435元，年均增长8%。城乡居民储蓄存款余额227.6亿元，是2000年的2.06倍；居民消费价格总水平保持基本稳定，年均上涨1.5%。

——和谐社会有序发展。社会治安综合治理工作被评为全国优秀，义马群众工作经验和渑池信访评估模式在全国推广。平安三门峡建设成效卓著，人民群众的安全感明显提高。

经过六年的不懈努力，我市经济社会发展站在了新的战略起点上，进入了全面优化产业结构、加快推进经济转型、促进统筹协调发展的新阶段。

回顾本届政府的工作，我们主要抓了以下五个方面：

（一）以项目建设为龙头，稳步推进工业化。我们咬定项目建设不放松，推进产业结构升级不松劲，加快培育能源、铝工业、煤化工、有色金属综合利用及深加工和林果生产加工等五大支柱产业，促进了资源优势向经济优势的转化。特别是2004年以来，全市新上投资3000万元以上的工业重点项目182个，总投资423亿元，是区划调整至“九五”末投资总和的1.15倍。五大支柱产业发展迅速，占全市生产总值的比重为42.5%。积极培育大型企业集团，全市销售收入超10亿元的企业达到8个，4家企业进入省百户重点企业行列。切实加强监测与指导，认真解决工业经济运行中的突出问题。不断加大融资力度，着力改善金融生态环境。大力发展循环经济，支柱产业链条进一步拉长，义马市被列为全国循环经济示范创建城市，9家企业被省政府认定为资源综合利用单位。

（二）加快新农村建设，推进农业现代化。高度重视“三农”工作，狠抓各项支农惠农政策落实，扎实推进新农村建设。全力完善农村基础设施，六年累计完成通油（砼）路行政村1209个，实现了道路、电话“村村通”；城乡电网改造全面完成，新发展沼气2.1万户，建成饮水工程5621处，解决了43万人的饮水安全和饮水困难。始终坚持用工业理念发展现代农业，积极推进农业结构调整，粮食产量突破6亿公斤，果、牧、林支柱产业占农业总产值的比重达到83%。大力发展农业产业化，全市龙头企业达到99个，湖滨果汁等3家企业被确定为省重点龙头企业。加快推进农业标准化，制定农业地方标准168项，灵宝大枣、卢氏鸡等6种农产品荣获国家“地理标志产品”称号。“三电一厅”建设加快，农业信息化走在全国前列。劳务经济迅速发展，六年累计输出劳动力100万人次。

（三）实施中心城市带动，提升城镇化水平。大力整治生态环境，完善城市功能，方便居民生活，努力建设最宜人居的自然山水城市。运三高速公路项目前期进展顺利，国道310、209线市域段改建完工，省道322、314、318线建设稳步推进，郑西铁路客运专线加快建设，覆盖全市的交通主框架初步形成。陕州大道建成通车，涧河治理全线蓄水，三门峡广场、湖滨广场建成开放，城市集中供热一期完成，集中供气、城市饮水工程全面启动，市、县污水处理厂全部投入使用。天鹅湖、上阳苑、涧河公园等景区（点）成为市民的休闲场所。扎实推进“四城联创”，顺利通过省级卫生城市和园林城市验收，环保模范城市、文明城市创建成效明显，城市品位和形象显著提升。310国道沿路城镇经济隆起带发展规划付诸实施，义马、渑池城区对接取得实质性进展，灵宝市北区建设初具规模，卢氏县新区开发初显成效。县域经济快速发展，3个县（市）跨入全省综合经济实力20强。

（四）全面深化各项改革，不断扩大对外开放。坚持以改革为动力，以开放促发展，激发经济社会发展活力。国有企业改革基本结束，财税体制改革逐步深化，部门预算、国库集中支付和政府采购制度全面实施。农村综合改革迈出重要步伐，农民负担连年保持全省最低水平。乡镇机构改革圆满完成，通过撤并共减少乡（镇）14个。行政审批制度改革不断深化，率先在全省实行投资项目核准、备案制，市级审批项目大幅削减。粮食流通体制和农村信用社等改革走在全省前列，投融资体制改革取得阶段性成效。交通、城建、水利、科技、教育、文化、价格、住房等改革迈出了新步伐。

积极实施开放带动战略，不断优化投资环境，外向型经济快速发展，中国大唐、英国开曼、东方希望等一批国内外知名企业落户三门峡，湖滨果汁、天元铝业和灵宝黄金股份公司在境外成功上市。成功举办了六届旅游节暨投洽会，虢国博物馆和豫西大峡谷被评为国家4A级旅游景区，旅游收入连年大幅增长。商贸交通、现代物流、文化、房地产、信息等第三产业健康发展。

（五）坚持以人为本，促进社会和谐。切实把民生问题、社会事业发展摆上重要位置，着力解决人民群众最关心、最直接、最现实的利益问题。认真做好就业和社会保障工作。六年城镇新增就业14.8万人，下岗失业人员再就业7.5万人。社会保险扩面、基金征缴工作成效显著，养老、失业、医疗基金征缴率均达到100%，企业职工基本养老保险实行市级统筹。六年累计发放城市低保资金1.43亿元。医疗救助制度覆盖城乡，农村低保制度全面推开。大力发展各项社会事业。六年累计组织实施市级以上科技项目707项，改造农村中小学危房29.5万平方米，34.13万人次享受到国家“两免一补”政策。文化、体育事业健康发展，“豫西文化长廊”建设全面启动，陕县和灵宝市被列入全国首批“农民体育健身工程试点县”。公共卫生体系不断完善，新型农村合作医疗深入开展，试点县（市）农民参合率达89.7%。扶贫开发力度不断加大，六年累计解决9.26万农村贫困人口的温饱问题。大力开展土地“三项整治”，较好地保障了建设用地需要。加强对煤炭、铝土矿的资源整合，实现了资源的集约利用。切实加强精神文明和民主法制建设。深入开展“三好一强”和保持共产党员先进性教育活动，公务员宗旨意识进一步增强，政府机关自身建设不断改进。深化精神文明创建活动，“双创”和“两抓一促”活动深入开展，市民文明素质稳步提高。坚持定期向人大及其常委会报告工作，向政协通报情况，执行人大及其常委会的决议，自觉接受法律和民主监督。六年来，认真办理了人大代表建议538件，政协委员提案1611件。巩固和扩大基层民主，顺利完成第四、第五届村民委员会换届选举。“四五”普法和依法治市全省领先。民兵预备役建设不断加强，双拥模范城创建荣获全国二连冠、全省五连冠。妇女儿童工作受到国家表彰。认真落实安全生产措施，安全形势基本稳定。深入开展商业贿赂专项治理，反腐倡廉工作取得新成效。统计、审计和经济监督工作进一步加强，新闻出版、民族宗教、防震减灾、外侨、对台、老龄、档案、农机、气象、史志、无线电、人防、残疾人救助等各项事业也取得了新进展。

各位代表，过去的六年，是一心一意谋发展、关注民生促和谐的六年，是经济社会全面进步、人民生活明显改善的六年，更是为实现再造一个三门峡、跻身中原崛起第一梯队宏伟目标奠定坚实基础的六年。这些成绩的取得，归功于省委、省政府和市委的正确领导，归功于市人大、市政协的监督支持，归功于全市广大干部群众的团结奋斗。在此，我代表市人民政府，向工作在全市各条战线上的工人、农民、知识分子、干部、人民解放军指战员、武警官兵、公安民警以及各界人士致以崇高的敬意和衷心的感谢！

在肯定成绩的同时，我们也清醒地看到，我市经济社会发展中仍然存在着一些深层次矛盾和问题：产业产品结构不尽合理，优化经济结构、转变增长方式的任务还很繁重；“三农”问题比较突出，新农村建设的任务依然艰巨；一些社会不稳定因素仍然存在，维护稳定的压力还比较大；部分群众的生活还比较困难，就业、医疗、教育、住房和社会保障等关系群众切身利益的问题还比较多；政府职能转变相对滞后，行政成本还比较高，机关作风建设还存在不尽如人意的地方等。对此，我们一定高度重视，在今后的工作中认真加以解决。

二、今后五年政府工作的指导思想、奋斗目标和基本要求

今后五年，政府工作的指导思想是：坚持以邓小平理论和“三个代表”重要思想为指导，深入贯彻落实科学发展观，紧紧围绕再造一个三门峡、跻身中原崛起第一梯队的奋斗目标，坚定不移地走科学发展、和谐发展之路，进一步突出优化经济结构和转变增长方式这一主线，以深化改革、扩大开放为动力，以维护稳定、构建和谐社会为前提，以加强政府自身建设为保障，奋力推进工业化、城镇化和农业现代化，努力建设经济发达、政治清明、文化繁荣、社会和谐、人民富裕、环境优美的新三门峡。

按照上述指导思想，今后五年的主要奋斗目标是：

——综合经济实力再上新台阶。主要经济指标超过全省平均增长水平，地区生产总值翻一番达到800亿元，地方财政一般预算收入翻一番以上超过45亿元，基本建成惠及全市人民更高水平的小康社会。

——经济增长方式实现新转变。集约发展、循环发展成效明显，支柱产业优势更加突出，资源综合利用率显著提高，单位生产总值能耗降低20%左右，生态环境更加宜人，高新技术产业、现代服务业比重有较大提高。

——城乡协调发展取得新突破。中心城市品位进一步提升，集聚、辐射和带动功能明显增强，成为全省发展潜力大、增长速度快、综合实力强的城市之一；县域经济发展进一步加快，建成一批社会主义新农村示范村，城市现代化和城乡一体化取得显著成效。

——人民生活质量得到新提高。城镇居民人均可支配收入和农民人均纯收入年均增长高于全省平均水平，物质生活更加富裕，精神更加充实，居住、交通、通信、教育、文化、卫生和环境等方面的条件有较大改善。

——和谐社会建设跨入新阶段。社会主义民主更加完善，社会主义法制更加完备，社会更加安定有序；社会事业全面发展，城乡文明程度进一步提高；社会保障体系更加健全，社会就业更加充分，公共服务更加有效。

做好今后五年的政府工作，要进一步增强发展意识，把握好发展方向和着力点，坚持以下五项基本要求：

一是坚持在提高质量的基础上推动快速发展。把“好”摆在更加突出的位置，注重优化结构，提高质量和效益，实现协调发展、平稳发展、持续发展，发展求好，好中求快，保持全市经济健康快速发展的良好态势。

二是坚持在落实宏观调控政策中推动转型发展。按照中央提出的“四个着力”，把宏观调控作为重要机遇，深刻领会中央精神，吃透上情，研究市情，乘势而上，顺势而为，在调控中谋求发展，在发展中落实调控，大力推进经济结构调整，促进增长方式转变。

三是坚持在改革开放中推动创新发展。围绕完善社会主义市场经济体制，加大重点领域和关键环节的改革力度，着力构筑落实科学发展观的体制保障。进一步确立新的开放观，实施新的开放举措，开创新的开放格局，不断提高对外开放的质量和水平。增强区域自主创新能力，努力建设创新型三门峡。

四是坚持在统筹兼顾中推动协调发展。坚持以经济建设为中心，在促进经济快速增长，不断增加物质财富的同时，更加注重发展的协调性和均衡性。积极采取有效措施，以工促农，以城带乡，实现新农村建设和城镇化联动发展，工业和农业竞相发展，经济与社会协调发展。

五是坚持在解决民生问题中推动和谐发展。牢记群众利益无小事，高度关注民生，不断完善为民办实事的长效机制，着力解决好人民群众最关心、最直接、最现实的利益问题，让人民群众见到实效，共享改革和发展成果。

三、2007年政府工作的主要目标和任务

今年全市经济和社会发展的主要预期目标是：全市生产总值增长13%；全社会固定资产投资增长19%；地方财政一般预算收入增长15%；社会消费品零售总额增长14%；实际直接利用外资增长22%，出口创汇增长15%；居民消费品价格指数控制在3%以内；农民人均纯收入增长5%以上，城镇居民人均可支配收入增长9%；单位生产总值能耗降低4.9%；化学需氧量、二氧化硫排放量分别减少3.5%和9.6%；新增城镇就业4万人，城镇登记失业率控制在4.5%以内；人口自然增长率控制在6.5‰以内。

围绕上述目标，重点抓好六个方面的工作：

（一）认真落实国家宏观调控政策，着力优化经济结构、转变经济增长方式

突出抓好重点项目建设。坚持项目带动战略不动摇，全面实施重大项目推进联动机制和项目建设目标管理责任制，确保开曼氧化铝三期、开祥化工甲醇、康耀导电玻璃二期、灵宝阿姆斯果蔬加工二期、龙王庄煤矿等一批已开工项目年内建成投产。争取天瑞30万吨铝铸件、鑫灵化工硫酸扩建等一批项目年内开工，完成建设进度。进一步加大项目前期工作力度，重点抓好大唐发电三期、义马煤制油、戴卡轮毂三期等一批重大项目前期工作，力争实现突破，保证项目接续。深入研究国家产业政策，结合五大基地建设，围绕新能源、新材料开发和新技术、新装备应用，超前论证谋划一批对经济社会发展有较强支撑的重大项目，充实项目储备，完善项目结构，备足发展后劲。继续抓好三门峡西工业园规划和建设，不断加大引资力度，拓宽招商渠道，力求项目建设取得实质性进展。全面推进郑西客运专线、运三高速公路等重大基础设施项目建设，按时完成计划进度。

加快推进工业结构调整。进一步调整投资方向，优化工业经济结构，积极争取结构调整专项资金，推进工业结构的战略性调整。围绕五大基地建设，拉长产业链条，壮大产业集群，扩大产业规模，进一步增强支柱产业的支撑能力。加强企业创新平台建设，运用先进适用技术和高新技术改造传统产业，加快资源型工业向制造型工业的转变。大力发展生物工程、医药、新材料、新能源等高新技术产业。利用国家优惠政策，重点开发电子玻璃、超高功率电极等高技术含量的载能产品。积极引导产业相近、产品同类企业重组、并购或联合，实现优势互补；全面实施名牌战略，大力培育产业链条完整的大型骨干企业集团，力争今年销售收入超50亿元的企业达到2个，超10亿元的达到10个以上，壮大我市特色工业的核心竞争力。

积极协调工业经济运行。完善监测预警手段，搞好对重点行业、重点产品产销情况的分析，建立完善产品质量检测体系，确保企业增产增效。做好煤炭产销衔接和电煤供应，保持煤炭供应的动态平衡。大力开拓电力市场，进一步加强发、输、用各环节的协调，争取骨干电厂效益稳步提升。按照国家产业政策，通过政府引导、企业自主，积极搭建高载能企业和火电企业联营发展的平台，实现互利双赢。加快建立现代企业制度，强化企业内部管理，提升经营管理水平。继续加强银政、银企协作，优化金融生态环境，引导金融机构增加贷款规模，支持地方经济发展。

加快发展现代服务业。认真落实加快服务业发展的政策措施，进一步加大指导和扶持力度，全面提高服务业比重和水平。搞好旅游资源开发及基础设施建设，重点抓好豫西大峡谷、仰韶大峡谷、卢氏双龙湾等景区基础设施的配套建设，提升综合服务能力；加强旅游行业管理，强化宣传促销，打造“文化山水”旅游品牌。以专业化和现代化营销手段改造提升传统服务业，积极拓展服务领域。推进现代物流业发展，加快培育现代物流企业，打造连锁经营、特许经营和电子商务流通平台。支持和培育金融保险、法律服务、会计审计事务等中介组织，大力发展房地产、信息技术、社区服务等新兴服务业，构筑高增值、多层次、广就业的现代服务体系，努力培育新的经济增长点。

（二）大力发展农村经济，扎实推进社会主义新农村建设

坚持发展特色农业。落实中央一号文件精神，大力发展现代农业。稳定粮食种植面积，努力提高单产和质量。加快特色农产品生产加工基地建设，做大做强支柱产业，确保果、牧、林等支柱产业产值占农业总产值的比重稳步提高，果品产量达到115万吨，肉、蛋、奶产量分别提高8%、7%、19%。积极推进农业产业化经营，不断壮大龙头企业，力争缘份果业、张村肉牛等企业进入省级农业龙头企业行列。进一步完善林权制度改革，完成人工造林25万亩，壮大林板一体化和果品深加工规模，促进林业经济发展。充分发挥资源和自然地理优势，加快中药材产业发展，种植面积达到22万亩，野生资源保护基地达到30万亩。积极推广烟田标准化管理，实施烟水配套，改善烟叶生产基础设施，切实提高烟叶质量。

稳步提高农业综合生产能力。以“五级联创”活动为载体，全面提高农业标准化水平。完善“三电一厅”服务功能，重点推进信息化向乡、村延伸。实施“科技入户”工程，大力推广先进适用的农业技术，积极运用现代科技改造传统农业。加快农业机械化步伐，提高农业装备水平。抓好县级动物检疫站和乡级兽医站建设，进一步完善动物防疫体系。加强自然灾害监测预报，提高抗灾、减灾能力。

继续加快新农村建设步伐。坚持实行工业反哺农业、城市支持农村的方针，多予、不取、放活，全面落实各项惠农政策，加快新农村建设进程。以发展农村经济、增加农民收入为重点，以“六有、六通、六化”为目标，科学规划、分类指导，深入开展“抓试点、争致富、办实事”活动，突出抓好50个市、县示范村建设，积极探索符合实际的新农村建设模式。建立现代农业投入保障机制，固定资产投资和政府土地出让收入要向农村倾斜，加快农村道路、安全饮水、沼气、卫生、教育等基础设施建设，改善农村生产生活条件。加快干线公路和县乡公路建设，确保省道314、318线建成通车。深入实施“户户通电”工程，全部消除“无电户”。推广生态农业模式，新增农村沼气用户2万户。搞好农田水利基本建设，争取窄口灌区节水改造和朱乙河水库除险加固工程开工。治理水土流失面积80平方公里，新增有效灌溉面积、旱涝保收田面积、节水灌溉面积、坡改梯面积各3万亩，解决安全饮水10万人。

切实增加农民收入。拓宽培训渠道，培养新型农民，打造劳动力品牌。培训农村劳动力10万人次，转移总量27万人次，实现劳务收入15亿元。加大扶贫开发整村推进力度，巩固和解决1.49万贫困人口的温饱问题，实施搬迁扶贫651户2677人。稳定农村土地承包经营体制，依法规范土地流转行为。认真落实大中型水库移民后期扶持政策，做好扶持资金发放工作，重点抓好窄口水库移民计划项目落实。认真落实政策，支持老区建设。切实加强涉农收费和价格管理，防止农民负担反弹。

（三）切实加快城镇化建设步伐，带动城乡区域协调发展

完善中心城市功能。坚持中心城市带动战略，围绕创造最宜人居和最佳发展环境的目标，以城市总体规划为指导，尽快编制完成城市详规，完善城市规划体系。抓好城市基础设施建设，新建上官路、茅津北路，改造甘棠路、河堤北路，提高道路通行水平。实施涧河五期治理，高起点建设城市湿地公园，提升城市重点景区的品位和层次。加快卫家磨水库复建工程进度，确保城市引水工程主体年底完工。稳步扩大集中供气、供暖覆盖面，积极实施城市生活垃圾无害化处理。扎实推进“城中村”改造，加强社区建设和管理，不断完善城市服务功能。深入开展“四城联创”活动，园林城市创建完成年度计划，卫生城市创建全面完成集贸市场和“五小”单位改造，环保模范城市创建突出抓好30项指标的落实，文明城市创建加快向前推进。

大力推进副中心城市和小城镇建设。进一步加快310国道沿路城镇经济隆起带建设，壮大产业规模，提升聚集水平。义马—渑池城区要继续围绕培育壮大能源、煤化工和铝工业等主导产业，加快推进城区对接。灵宝市要充分发挥有色金属综合利用、林果业生产加工的优势，进一步提升辐射带动能力。卢氏县要深入挖掘生态旅游资源，搞好基础设施建设，培育比较优势明显、特色鲜明的支柱产业。积极推进城镇户籍制度改革，着力支持重点城镇发展，切实发挥小城镇在经济、文化等方面的辐射功能。引导社会资金投入小城镇建设，促使经济要素向城镇集聚。

加快发展民营经济、县域经济和乡镇经济。坚持把民营经济作为县域经济发展的突破口，进一步用足、用活、用好国务院“非公经济36条”，激发民营资本活力。鼓励引导县域发展各具特色的工业聚集区，承接产业转移，增强综合实力。全力支持义马市冲刺“全国百强县”，确保灵宝市、渑池县在全省的位次稳步前移，力争陕县尽快进入全省20强，卢氏县进入全省10快。进一步落实和完善乡镇经济社会发展检测评价办法，健全考核和激励机制，充分调动各乡镇加快发展的积极性，努力使乡镇经济真正成为壮大县域经济、建设社会主义新农村的重要基石。

（四）不断扩大改革开放，着力优化经济发展环境

扎实推进重点领域的改革。继续深化国有企业改革，不断完善企业法人治理结构、投资风险控制机制和内部监督管理机制，确保国有资产保值增值。继续深化财政体制和预算管理制度改革，全面加强税源建设，完善公共财政体系，提高收入质量和资金使用效益，建设“阳光财政”。认真实施事业单位收入分配制度改革，调整优化支出结构。巩固农村税费改革成果，全面推进以乡镇机构、农村义务教育、县乡财政管理体制为主要内容的农村综合改革。继续深化文化体制改革，加大经营性文化事业单位转企改制力度，大力发展民营文化产业。同时，扎实推进粮食流通、公用事业、医疗卫生等领域的改革，力争取得阶段性成果。

大力发展外向型经济。积极参加全省组织的各类商贸洽谈活动，多形式、多渠道引进和利用市外资本。不断创新招商方式，重点围绕五大支柱产业和优势资源推介项目。继续加大对签约项目的跟踪落实力度，提高招商引资的质量和水平。充分利用“两个市场”，优化出口商品结构，着力培育糖化酶、果汁饮料、铜箔等新的出口品种，扩大机电产品和高新技术产品出口，提高出口效益。

不断优化经济发展环境。综合运用行政、法律、经济和舆论等手段，优化政策环境、法治环境、服务环境、人文环境和信用环境，着力建设优化环境的长效机制。加大企业周边和重点项目建设环境治理力度，整顿和规范市场经济秩序，强化市场流通、经营秩序等环境的综合治理，营造行为透明、办事高效、服务优良的宽松环境，让投资者放心满意，让落地项目生根结果。大力整治教育乱收费和公路“三乱”，加大商业贿赂专项治理力度，坚决纠正损害群众利益的不正之风。

（五）着力加强资源节约和环境保护，提高集约节约发展水平

积极推进节能降耗工作。建立健全节能降耗目标责任制，完善重点用能单位能源利用报告制度。依靠科技进步，改造黄金、水泥、电解铝等高耗能行业和企业，大力推广节能降耗新技术，重点支持节能、节水和资源综合利用项目建设，推进“三废”综合利用，逐步推广城市中水利用，提高资源节约和综合利用效益。积极开发风能、水能等清洁能源，加快推进黄河风电项目建设。发展新型墙体材料，抓好建筑、交通、商业和政府机构等领域的节能降耗工作。

大力发展循环经济。认真编制全市循环经济发展规划，积极开展全国循环经济试点城市创建工作。围绕优势产业，加快粉煤灰、煤矸石和各种伴生矿的综合利用，逐步拉长产业链条，实现清洁发展、循环发展和可持续发展。鼓励各类企业认真落实政策措施，努力发展循环生产。积极推进农村废弃物的资源化、减量化、无害化，探索农产品基地与加工企业之间的循环发展新模式。

提高土地和矿产资源集约节约利用水平。实行严格的土地管理制度，新增建设用地主要用于保障省、市重点项目建设需要。以工业项目聚集区为重点，执行用地定额标准，规划多层标准厂房，提高单位土地面积的投资强度。进一步加大土地开发复垦整理力度，深入开展“三项整治”活动，努力实现耕地占补平衡。巩固煤炭、铝土矿资源整合成果，坚决关闭破坏环境、污染严重、不具备安全生产条件的矿山企业，规范矿产开发秩序，提高资源开发利用水平。

加强环境保护和生态建设。深入开展环保六大专项行动，重点解决影响群众身体健康的环境问题，努力完成减排降污的各项目标任务。强力推进电厂脱硫，确保所有火电机组年内全部实现脱硫排放。全面落实环境影响评价、污染物排放总量控制和环保目标责任制，加强污染源综合治理。严格执行新上项目环保“三同时”，杜绝产生新的污染。妥善做好辖区内重点流域、区域和重点企业的污染防治。继续实施城郊大环境绿化工程，抓好重要通道两侧的荒山荒坡、农田林网以及沿黄滩涂的造林绿化。扎实开展林业生态县、乡、村三级创建活动，建设绿色三门峡。

（六）积极促进社会发展和解决民生问题，扎实推进和谐社会建设

认真做好再就业和社会保障工作。全面落实就业再就业扶持政策，重点做好下岗失业人员再就业工作，积极帮助“零就业”家庭实现就业。高度重视大中专毕业生就业问题。进一步健全社会保障体系，大力开展社会保险扩面征缴工作，全面落实各项社会保险待遇。逐步完善城市低保制度，实现动态管理下的应保尽保。高度关注进城务工人员子女入学、医疗保险等问题，认真清理建设领域拖欠工程款和农民工工资，维护进城务工人员合法权益。扎实落实农村低保制度，逐步提高补差标准。加快农村敬老院建设，农村五保集中供养率提高到30%以上。加大经济适用房和廉租房开发力度，认真解决城镇困难群众住房难问题。

全面发展社会事业。积极实施科技发展中长期规划纲要，优化科技资源配置，加强交流与合作，注重人才的引进和培养，建设创新型三门峡。大力实施素质教育，努力实现基础教育、职业教育和成人教育协调、均衡发展。整合中小学教育资源，开展教师岗位培训，提高教育教学水平。全部免除农村义务教育阶段学生的学杂费，继续对农村和市区困难家庭学生免费提供教科书并补助寄宿生活费。稳步提高农村中小学公用经费保障水平，改善农村中小学教育教学设施。围绕“豫西文化长廊”建设规划，加大对公益性文化事业的投入。加强文物保护和利用，做好北阳平遗址列入国家“十一五”大遗址保护项目的相关工作。继续实施广播电视“村村通”和农村电影放映工程，加快农村文化中心和文化大院建设。大力开展城市社区卫生服务，全面实行新型农村合作医疗。逐步改造乡镇卫生院，有效改善农村医疗卫生条件。继续稳定低生育水平，努力降低出生人口性别比，提高人口素质。加强食品药品监管和物价、统计、无线电、民族宗教等工作，重视妇女儿童、残疾人事业，大力发展慈善事业，继续做好外侨、对台、老龄、史志、档案等工作。

切实抓好安全生产。认真贯彻“安全第一，预防为主，综合治理”的方针，全面落实安全生产责任制，突出搞好煤矿、非煤矿山、危险化学品、交通运输、烟花爆竹、食品药品、消防安全等重点领域专项整治工作，加强学校和人员密集场所安全防范。特别要建立健全煤矿安全监管工作长效机制，坚决遏制重特大安全事故发生，促进安全生产整体形势稳定好转。

继续加强社会治安综合治理。完善社会治安防控体系，广泛开展平安创建活动，保障人民群众安居乐业。积极落实信访责任制，认真解决群众反映强烈的热点、难点问题，将矛盾消除在基层、化解在萌芽状态。加快应急体系建设，积极预防和妥善处置各类突发事件，努力提高公共安全保障能力，维护社会大局稳定。

高度重视精神文明和民主法制建设。切实加强理想信念教育，实施公民道德建设工程，不断提高公民素质。继续开展“三理”教育，积极开发青少年儿童教育基地，加强和改进未成年人思想道德建设。深入推进“人人树形象，增辉三门峡”活动，建设诚信三门峡。繁荣发展新闻出版、广播影视、文学艺术和哲学社会科学。广泛开展群众性文明创建活动，扎实组织文化、科技、卫生“三下乡”，继续抓好科教、文体、法律、卫生“四进社区”，不断提高城乡文明程度。严格执行人大及其常委会的各项决议和决定，自觉接受人大及其常委会的法律监督和人民政协的民主监督。认真办理人大代表、政协委员的建议和提案，广泛听取各民主党派、工商联和无党派人士的意见和建议，充分发挥工会、共青团、妇联等群众组织的桥梁纽带作用。加强基层民主建设。坚持依法治市，深入开展“五五”普法教育，提高全民法律素质。加强法律援助，保障公民特别是弱势群体的合法权益。

积极支持部队和国防建设。深入开展国防教育，关注和支持驻市解放军和武警部队建设。抓好人民防空和民兵预备役工作。认真落实军人优待抚恤政策，做好军队转业干部、复员退伍军人接收安置工作。深入开展双拥共建活动，巩固和发展军政军民团结，争取“全国双拥模范城”实现三连冠。

四、加强政府自身建设

按照“为民、务实、清廉”和树立“八个方面良好风气”的要求，大力开展“讲正气、树新风、谋发展、促和谐”活动，切实加强政府自身建设，做到为人民服务、对人民负责、受人民监督、让人民满意。

执政为民，建设服务型政府。坚持人民利益高于一切，把实现好、维护好、发展好人民群众的根本利益作为政府工作的出发点和落脚点，千方百计促民富，尽心竭力解民忧，忠于职守保民安。转变服务观念，改进服务方式，提高服务质量，更好地为基层、企业和社会公众服务。积极整合行政资源，有效降低行政成本，切实提高行政效率。大力推进政务公开，在公共服务部门和单位全面推行办事公开制度。积极推进电子政务建设，建立和完善政府门户网站，适时启动政务外网工程。健全行政服务大厅功能，精简办事程序，切实履行服务承诺。认真解决土地征用、房屋拆迁和企业改制中损害群众利益的突出问题，做到察民情、解民忧，言必行、行必果，切实取信于民。

改革创新，建设效能政府。以转变政府职能为重点，推进行政管理体制改革，实行政企分开、政资分开、政事分开、政府与市场中介组织分开。继续深化行政审批制度改革，进一步减少和规范行政审批事项。搞好经济调节和市场监管，更加注重社会管理和公共服务。依法管理和规范社会组织、社会事务，协调各种利益关系，促进社会公平和正义。完善政府责任目标考评制度，增加衡量科学发展、和谐社会建设等指标，实施GDP能耗指标公报制度。增强改革主动性，以改革促发展，向改革要效益。

依法行政，建设法治政府。深入贯彻《行政许可法》和《全面推进依法行政实施纲要》，进一步提高依法行政水平。加强政府法制建设，全面落实行政执法责任制，明确行政执法主体，规范行政执法行为。依法搞好行政复议，加强行政执法监督。健全重大事项和涉及群众切身利益事项的决策公示、听证制度，推进决策科学化、民主化。严格执行行政问责制，加强行政效能监察，提高政府执行力和公信力。全面推行领导干部经济责任审计制度。严格执行项目招投标、土地“招拍挂”、国有资产转让、政府采购等制度，防止违法违纪行为发生。

勤俭节约，建设廉洁政府。牢记“两个务必”，带头厉行节约，反对奢侈浪费。规范公务接待，严禁公款吃喝，严格控制公款出国，严格控制行政机关新建、扩建办公大楼，堵塞管理漏洞，降低行政成本，建设节约型机关。落实廉政建设责任制，深入开展廉政建设和反腐败斗争，完善教育、制度、监督并重的惩治和预防腐败体系。

真抓实干，建设务实政府。树立正确的政绩观，坚持求真务实，多做打基础、备后劲、管长远的工作，克服官僚主义和形式主义。继续大力精简会议、文件，坚决改进会风、文风，严格控制和规范各种检查、评比、达标活动。大兴调查研究之风，掌握真实情况，解决实际问题。强化工作责任，加强督促检查，狠抓工作落实。认真实施《公务员法》，加强公务员教育、培训和管理，建设一支高素质的公务员队伍。

各位代表，回首过去，我们感到欣慰和自豪；展望未来，我们充满信心和希望！让我们在省委、省政府和市委的正确领导下，高举邓小平理论和“三个代表”重要思想伟大旗帜，认真落实科学发展观，同心同德，锐意进取，为建设富裕、文明、和谐、美好的三门峡而努力奋斗！
